# Analgesic, Anti-Inflammatory, and Chondroprotective Activities of *Cryptolepis buchanani* Extract: *In Vitro* and *In Vivo* Studies

**DOI:** 10.1155/2014/978582

**Published:** 2014-08-27

**Authors:** Nutthiya Hanprasertpong, Supanimit Teekachunhatean, Rujirek Chaiwongsa, Siriwan Ongchai, Puongtip Kunanusorn, Chaichan Sangdee, Ampai Panthong, Samreang Bunteang, Narong Nathasaen, Vichai Reutrakul

**Affiliations:** ^1^Department of Pharmacology and Center of Excellence for Innovation in Chemistry, Faculty of Medicine, Chiang Mai University, Indhawaroros Road, Chiang Mai 50200, Thailand; ^2^Department of Medical Technology, Faculty of Associated Medicine, Chiang Mai University, Indhawaroros Road, Chiang Mai 50200, Thailand; ^3^Thailand Excellence Center for Tissue Engineering and Stem Cells, Department of Biochemistry and Center of Excellence for Innovation in Chemistry, Faculty of Medicine, Chiang Mai University, Indhawaroros Road, Chiang Mai 50200, Thailand; ^4^Department of Chemistry and Center of Excellence for Innovation in Chemistry, Faculty of Science, Mahidol University, Rama VI Road, Bangkok 10400, Thailand; ^5^The Forest Herbarium National Park, Wildlife and Plant Conservation Department, Ministry of Natural Resources and Environment, Pholyothin Road, Bangkok 10900, Thailand

## Abstract

*Cryptolepis buchanani* Roem. & Schult. is widely used in folk medicine in Southeast Asia for treating muscle tension and arthritis. This study aimed to investigate an analgesic activity of the methanol extract of *C. buchanani* (CBE) in acetic acid-induced writhing response in mice, and to examine its anti-inflammatory activity in ethyl phenylpropiolate- (EPP-) induced ear edema and carrageenan-induced paw edema in rats. Its effects on cartilage degradation induced by interleukin-1*β* (IL-1*β*) in porcine cartilage explant culture were also determined. This study demonstrated that CBE significantly reduced acetic acid-induced writhing response. It also inhibited edema formation in both EPP-induced ear edema and carrageenan-induced paw edema models. In cartilage explant culture, CBE significantly reduced the sulfated glycosaminoglycan and hyaluronan released into culture media while it reserved the uronic acid and collagen within the cartilage tissues. It also suppressed the matrix metalloproteinase-2 activity with no effect on cell viability. In conclusion, CBE shows analgesic, anti-inflammatory, and chondroprotective effects in this preliminary study. Therefore, CBE may be useful as an alternative treatment for osteoarthritis.

## 1. Introduction

Osteoarthritis (OA) is a chronic degenerative disorder characterized by destruction of articular cartilage and periarticular bone remodeling [1]. A World Health Organization report on the global burden of disease indicates that knee OA is likely to become the fourth most important global cause of disability in women and the eighth most important in men [2]. Because of the higher prevalence of OA, it is a more costly disease than rheumatoid arthritis in economic terms [3].

In OA, degradation of extracellular matrix component and consequent gradual articular cartilage destruction is caused by an increase in cartilage catabolism and a decrease in cartilage anabolism [4]. Clinically, this cartilage pathology is often accompanied by pain and joint dysfunction. Pain in and around the joint is the first and predominant symptom of OA that makes the patient seek treatment. Some patients also have inflammatory flares characterized by the episodic synovial effusion and increased pain and morning stiffness [5]. Goals of managing OA include controlling pain, maintaining and improving the range of movement and stability of affected joints, and limiting functional impairment [2, 6]. Approximately one-third of direct OA cost is allotted for medications, much of which goes toward the analgesic agents [3]. However, many patients suffer from a peptic ulcer side effect of the synthetic analgesic and anti-inflammatory agents such as nonsteroidal anti-inflammatory drugs (NSAIDs). The intensive searching for alternative drug with analgesic, anti-inflammatory, and chondroprotective properties especially from natural sources such as herbs for the treatment of OA is an important research effort.

*Cryptolepis buchanani* Roem. & Schult. (Asclepiadaceae) is a climbing tree found in evergreen forest in Thailand, China, India, Nepal, and Indo-China. It is widely used in folk medicine in Southeast Asia [7]. In Thailand,* C. buchanani* known as “Thao En On” has been used for treating inflammatory conditions such as muscle and joint pain [7–10]. Its stems are used in the treatment of muscle tension, stiffness of tendon, and arthritis [8, 10]. Its leaves are used as poultice on inflamed area for the treatment of myalgia and arthritis [10]. Few studies have examined the anti-inflammatory effect of this plant and found that its extract could reduce inflammation both in* in vitro* and* in vivo* studies [7, 11]. However, scientific reports of the analgesic and chondroprotective activities of* C. buchanani* are limited. The objectives of this study were therefore to evaluate the analgesic and anti-inflammatory activities of* C. buchanani* methanolic extract (CBE) in animal models and its chondroprotective effect in cartilage explants.

## 2. Materials and Methods

### 2.1. Chemicals

Carrageenan and ethyl phenylpropiolate (EPP) were obtained from Fluka Chemical Co., Ltd. (USA). Acetic acid was purchased from Merck (Darmstadt, F.R. Germany). Indomethacin, chondroitin sulfate C, D-glucuronic acid lactone, hyaluronan (from human umbilical cord), matrix metalloproteinase-2 (MMP-2), and recombinant-human interleukin-1*β* were obtained from Sigma (Sigma Aldrich, St. Louis, MO, USA).

### 2.2. Plant Material and Extract Preparation

**Figure 1:**
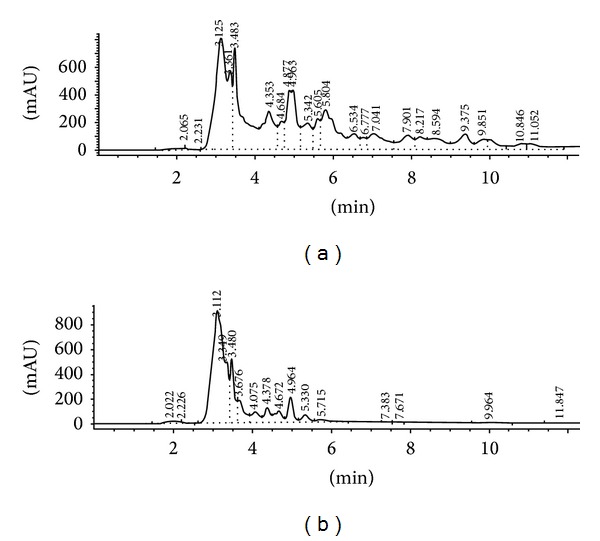
High-performance liquid chromatographic (HPLC) fingerprints of CBE. The chromatographic separation was carried out on a Fortis C18 (4.6 × 150 mm) column using a gradient elution consisting of water/methanol/acetonitrite (1/4/95, v/v) as a mobile phase. The UV absorbance was monitored with diode array detection (DAD) at 254 nm (a) and 320 nm (b).

The stems of* C. buchanani* were collected in Chiang Mai province and a voucher specimen BKF no. 137513 was deposited at the Forest Herbarium, Royal Forestry Department, Bangkok, Thailand. Dried and finely powdered stems, 3.85 kg, was percolated at room temperature with 4 × 64 L of methanol. The filtrate was evaporated under reduced pressure and freeze dried to give 452 g of the crude extract. The HPLC fingerprints of CBE were shown in Figure 1.

### 2.3. Animals

Male Spraque-Dawley rats weighing 40–60 g and 100–120 g as well as male Swiss albino mice weighing 30–40 g were purchased from the National Laboratory Animal Center, Nakhon Pathom, Thailand. All animals were kept in a room maintained under environmentally controlled conditions of 24 ± 1°C and 12 h light-dark cycle. The animals had free access to water and food and were acclimatized at least one week before starting the experiments. All animals received the test drugs in an equivalent volume of 0.1 mL/10 g body weight. Control groups received the same volume of vehicle by the same route. All procedures involving the use of animals were approved by the Animal Ethics Committee, Chiang Mai University (protocol number 20/2551).

### 2.4. Acetic Acid-Induced Writhing Response in Mice [12, 13]

Male Swiss albino mice weighing 30–40 g were divided into 5 groups, 6 rats per group. Methanol extract of* C. buchanani* at doses of 60, 125, and 250 mg/kg or indomethacin at dose of 5 mg/kg were administered intraperitoneally 30 min before the acetic acid injection. The control group received 5% Tween 80 (vehicle). A writhing response, a surrogate of pain, was produced by an injection of an aqueous solution of 0.75% acetic acid in a volume of 0.1 mL/10 g body weight into the peritoneal cavity; the animals were then placed in a transparent plastic box. The number of writhes, a response consisting of contraction of abdominal wall, pelvic rotation followed by hind limb extension, was counted during continuous observation for 15 min beginning from 5 min after the acetic acid injection. The number of writhes was compared between the treatment and the control group.

### 2.5. Ethyl Phenylpropiolate- (EPP-) Induced Ear Edema in Rats [14]

Male Spraque-Dawley rats weighing 40–60 g were divided into 3 groups, 5 rats per group. Ear edema was induced by the topical application of EPP 20 *µ*L/ear (EPP in acetone at concentration 50 mg/mL) to the inner and outer surfaces of each ear by an automatic microliter pipet.

The extract and indomethacin at dose 1 mg/mL were dissolved in 5% DMSO in ethanol and applied topically in a volume of 20 *µ*L to inner and outer surfaces of the ear just before the irritant. The control group received 5% DMSO in ethanol only. Before and at 15, 30, 60, and 120 min after edema induction, the thickness of each ear was measure with digital vernier caliper. The increase in the ear thickness was compared between the treatment and control groups.

### 2.6. Carrageenan-Induced Hind Paw Edema in Rats [15]

Male Spraque-Dawley rats of 100–120 g body weight were divided into 5 groups, 6 animals per group. The extract at doses of 100, 250, and 500 mg/kg, indomethacin at dose of 10 mg/kg or vehicle (5% tween 80 in control group), were given intraperitoneally 30 min prior to carrageenan injection. Lambda carrageenan was prepared as 1% suspension in sterile NSS. A volume of 0.05 mL of 1% carrageenan was injected intradermally into the plantar side of the right hind paw of an unanesthetized rat restrained in a plastic cage to induce paw edema, a surrogate of inflammation.

Foot volume of animal was determined by means of a volume displacement technique using a plethysmometer (model 7150, Ugo Basile, Italy). The right hind paw was immersed into the measuring chamber containing 0.05% NaCl in distilled water, exactly to and ink mark at anatomical hair line. Each paw volume was obtained from the average of 3 readings. The paw volume was measured prior to and at 1, 3, and 5 h after carrageenan injection. The edema volume of the treatment groups were compared to that of the control group.

### 2.7. Cartilage Explant Culture [16]

Articular cartilage was dissected from the metacarpophalangeal joints of pigs aged 20–24 wk. Cartilage discs (3 mm^2^) were biopsied from the weight-bearing region of the articular surface. Randomly selected explant discs (3 per well, approximately 30 mg in total) were cultured in a 24-well tissue culture plate with serum-free medium, DMEM containing 200 units/mL penicillin and 200 *μ*g/mL streptomycin. The explants were maintained in a humidified incubator with 5% CO_2_ at 37°C. Normal control group was the group with no treatment. Recombinant human interleukin-1*β* (rHuIL-1*β*), 15 ng/mL, was added to culture media to induce cartilage degradation in the presence or absence of CBE. As a positive control, culture medium supplemented with diacerein was used. Treatments were performed with triplicated wells using tissue from the same animal donor. Conditioned media were collected on day 3 of culture and stored at −20°C until analysis.

### 2.8. Dye Binding Assay [17]

The sulfated glycosaminoglycan (s-GAG) concentrations were determined using a colorimetric dye binding assay modified by Farndale et al. The assay is based on a metachromatic shift in absorption as a complex compound is formed in a mixture of 1,9-dimethylmethylene blue (DMMB) and the s-GAG in the sample and standard. The dye solution was made by adding 16 mg of DMMB to 5 mL ethanol to 2 g of sodium formate and 2 mL of formic acid in a total volume of 1 L at pH 3.5. The maximum absorbance of the dye solution was at 620 nm. This solution was stored at 4°C in a dark bottle. Chondroitin 6-sulfate (CS-C) standards (0–40 *μ*g/mL: 50 *μ*L) or samples (50 *μ*L) were transferred to a microtitre plate. The dye solution (200 *μ*L) was added immediately to each well and absorbance was measured at 620 nm; a precipitate might form on standing. A standard curve of CS-C concentration and absorbance 620 nm was plotted. The concentration of CS-C in the sample was calculated from the standard curve.

### 2.9. A Competitive Inhibition-Based ELISA for Hyaluronan (HA) 

Microtiter plates (Maxisorp, Nunc) were coated at 4°C overnight with umbilical cord HA (100 *μ*L/well) in the coating buffer. Uncoated area was then blocked with 150 *μ*L/well of 1% (w/v) bovine serum albumin (BSA) in the incubating buffer for 60 min at 25°C. After washing, 100 *μ*L of the mixture, sample or standard competitor (HA Healon range: 39.06–10,000 ng/mL) in biotinylated HA-binding protein (1 : 100), was added. After incubation for 60 min at 25°C, plates were washed and then the peroxidase-mouse monoclonal antibiotin (100 *μ*L/well; 1 : 4,000) was added and incubated for 60 min at 25°C. The plates were washed again and then the peroxidase substrate (100 *μ*L/well) was added and incubated at 37°C for 20 min to allow the color to develop. The reaction was stopped by addition of 50 *μ*L of 4 M H_2_SO_4_. The absorbance ratio at 492/690 nm was measured using the Titertek Multiskan M340 multiplate reader.

### 2.10. Uronic Acid Assay [18–20]

Hexosamine and uronic acid are the components of the repeating units of all glycosaminoglycans (GAGs). Uronic acid is widely determined as the representative of GAGs in biological substances. This assay measures uronic acid content in the cartilages by the releasing of monosaccharide using acid hydrolysis. For a standard curve, 0 to 2.4 *μ*g of glucuronic acid lactone or sample (5 *μ*L of dilution; 1 : 10) in up to 60 *μ*L of water was added to a test tube. Concentrated sulfuric acid-borate reagent (300 *μ*L) was added to the tube and mixed. The tubes were then incubated at 100°C in a water bath for 10 min and cooled to room temperature in ice bath. The solution of carbazole 50 mg/40 mL absolute ethanol (12 *μ*L) was added and mixed. The uronic acid reaction was incubated at 100°C in a water bath for 15 min. The absorbance of the pink to red color was read by spectrophotometer at 540 nm against distilled water blank.

### 2.11. Collagen Content Measured by Hydroxyproline (HPR) Assay [21]

The collagen content in the cartilage was calculated based on HPR determinations. A portion of papain-digested sample and culture medium was hydrolyzed with 6 M HCl for 14 h at 90°C and 24 h at 60°C. Samples (50 *μ*L) were combined with 100 *μ*L freshly prepared oxidizing solution (0.178 g chloramines-T in 15 mL n-propanol/10 mL ddH_2_O/25 mL citrate buffer (50 g citric acid monohydrate, 12 mL glacial acetic acid, 120 g sodium acetate trihydrate, 34 g NaOH to pH 6.0 in 1 L)) and incubated for 5 min at room temperature. Samples were mixed with 0.5 mL Ehrlich's reagent (1 g* p*-dimethylaminobenzaldehyde in 20 mL n-propanol, 6.6 mL perchloric acid, 15.6 mL ddH_2_O) and incubated at 60°C for 45 min, and absorbance at 570 nm was read by spectrophotometer. Samples were extrapolated against HPR standards between 0 and 10 *μ*g/mL.

### 2.12. Gelatin Zymography [22]

This method was used to determine matrix metalloproteinase-2 (MMP-2) in tissue culture media. Its gelatinolytic activity was assayed by electrophoresis. Sodium dodecyl sulfate-polyacrylamide gel electrophoresis (SDS-PAGE) was performed using a vertical gel apparatus according to the method of Laemmli [23], with modification by which gelatin was included in the resolving gel. Gelatin type B (Sigma-Aldrich) was copolymerized at a final concentration of 1 mg/mL into 0.75 mm thick, 10% polyacrylamide gel. Samples were mixed with an equal volume of 2x sample buffer and incubated at room temperature for 30 min. Each sample was loaded to a well and the samples were electrophoresed for 200 min at 90 V. After electrophoresis, SDS was removed from the gel by washing 2 × 15 min in 2.5% Triton X-100 at room temperature and incubated for 16 h at 37°C in activating buffer. The gels were subsequently stained with 0.2% Coomassie Brilliant Blue R-250 for 5 h at room temperature and destained with 50% methanol and 10% acetic acid to reveal zone of lysis within the gelatin matrix. The gel was dried on a Whatman paper. Molecular weight standard markers were run on each gel. Gelatinolytic activity was detected as clear band against a background of stained intact gelatin-impregnated acrylamide gel.

Quantification of the gelatinolytic band on the zymogram was performed by densitometry. The image acquisition was done with an Agfa scanner (SNAPscan 1212), by using Adobe Photoshop Elements 2.0 program. The zymogram densitometry was achieved with an Acion Image software for PC (Scion Corporation, Frederick, Maryland, USA), working in the gel plot 2 mode.

### 2.13. Cell Viability Determined by Lactate Dehydrogenase (LDH) Assay [24]

Cell viability was assessed by measuring LDH activity via colorimetric assay. The increase of LDH activity in culture media was proportional to the number of cell lysis.

### 2.14. Statistical Analysis

The data from the experiments were expressed as mean ± S.E.M. Statistical comparison between groups was analyzed by using one-way analysis of variance and post hoc least-significant difference test. *P* value less than 0.05 was considered significant.

## 3. Results

### 3.1. Effect of CBE on Acetic Acid-Induced Writhing Response in Mice

All 3 doses of CBE significantly reduced the number of writhes compared with the control group (Table 1). However, the percentage of inhibition of writhing by CBE 125 mg/kg was similar to that of CBE 250 mg/kg. Indomethacin 5 mg/kg decreased the number of writhes significantly when compared with the control and CBE groups (*P* ≤ 0.01).

### 3.2. Effect of CBE on EPP-Induced Ear Edema in Rats

The mean changes of ear edema thickness are shown in Table 2. CBE and indomethacin both at the dose of 1 mg/ear significantly reduced ear edema formation induced by EPP when compared with that of the control group (*P* ≤ 0.001). There was no significantly different effect between CBE and indomethacin groups.

### 3.3. Effect of CBE on Carrageenan-Induced Hind Paw Edema in Rats

Indomethacin 10 mg/kg and CBE at doses 250 and 500 mg/kg significantly decreased the carrageenan-induced hind paw edema volume when compared with the control group at every time point (Table 3). Although CBE at the dose of 100 mg/kg tended to reduce paw edema volume but significant reduction was found only at the 3rd h. At the 3rd and the 5th h, CBE dose dependently and significantly reduced paw edema.

### 3.4. Chondroprotective Effect of CBE in Porcine Cartilage Explants

**Figure 2:**
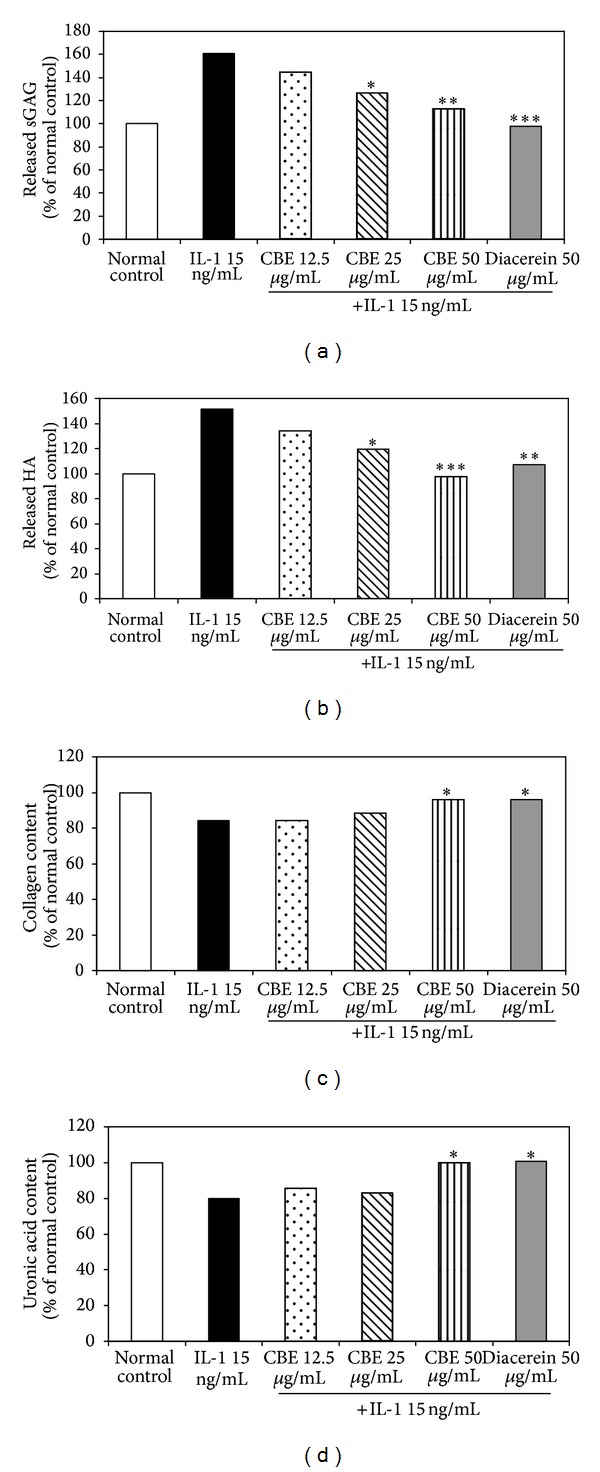
Effects of CBE on the release of s-GAG (a) and HA (b) into the medium as well as the content of uronic acid (c) and collagen (d) left in cartilage disc. Data are percentage of the control group. ∗, ∗∗, and ∗∗∗ denote significant difference from the IL-1*β* group (*P* < 0.05, *P* ≤ 0.01, and *P* ≤ 0.001, resp.).

After treatment for 3 days, IL-1*β* alone increased the release of s-GAG and HA into the culture media while significantly reduced uronic acid and collagen reserved in cartilage discs when compared with the normal control group (Figure 2). Coculture of IL-1*β* and CBE 50*μ*g/mL significantly reduced s-GAG and HA level in the culture media, while significantly reserved uronic acid and collagen content in cartilage discs in comparison to the treatment of IL-1*β* alone. But these effects were not different from those of the normal control group. The results of CBE 50 *µ*g/mL group were similar to those of diacerein 50 *µ*g/mL group.

**Figure 3:**
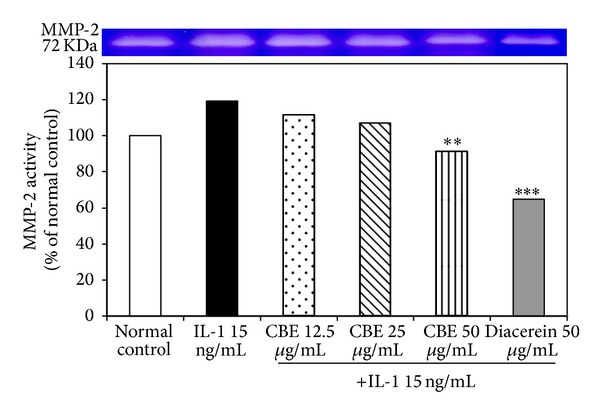
Effects of CBE on MMP-2 activity. ∗∗*P* ≤ 0.01 and ∗∗∗*P* ≤ 0.001 compared with IL-1*β* group.

IL-1*β* significantly increased the activity of MMP-2 whereas CBE 50 *µ*g/mL and diacerein significantly reduced MMP-2 activity (Figure 3). CBE 12.5 and 25 *µ*g/mL also decreased MMP-2 activity but the reduction did not reach statistical significance.

### 3.5. Cell Viability Determined by LDH Assay

All doses of the plant extract (6.25, 12.5, 25, 50, and 100 *µ*g/mL) did not affect cell viability when compared to the control group (data not shown).

## 4. Discussion

CBE significantly reduced the acetic acid-induced writhing response in mice. It inhibited the edema formation in both EPP-induced rat ear edema and carrageenan-induced rat paw edema. CBE significantly reduced the s-GAG and HA released from cartilage explants into the culture media while reserved the cartilage matrix molecules such as uronic acid and collagen. It also suppressed the MMP-2 activity with no effect on cell viability. CBE therefore exhibits promising analgesic, anti-inflammatory, and chondroprotective effects.

OA is a degenerative joint disease which is one of the most common arthritis. It is characterized by gradual loss of articular cartilage, hypertrophy of marginal bone (i.e., osteophytes), and subchondral bone sclerosis [25]. Pain management is the main indication for drug treatment in OA patients [26]. In folk medicine, stems of* C. buchanani* have been used to relief muscle and joint pain [8, 10]. In this study, we found that all doses of CBE given intraperitoneally could reduce the number of writhes in mice significantly when compared with the control group. The percentage of inhibition of CBE 125 and 250 mg/kg were similar. So CBE 125 mg/kg was probably a maximum analgesic dose. However, the analgesic efficacy of CBE was lower than indomethacin. A model of acetic acid-induced writhing response in mice is widely used as a screening analgesic model. Acetic acid possibly induces algesia by causing intra-abdominal tissues damage and release of pain-producing substances which excite pain nerve endings [12, 27]. These substances include H^+^, K^+^, histamine, bradykinin, prostaglandins (PGs), serotonins, and substance P [28]. Because this test lacks specificity [29], further tests should be performed to evaluate the mechanism of analgesic action of CBE.

Inflammation underlies the pathology in OA [30–32]. Human OA-affected cartilage expresses cyclooxygenase-2 (COX-2) and spontaneously produces PGE_2_. It also produces other cytokines and mediators associated with inflammation such as nitric oxide, IL-1*β* and tumor necrosis factor-*α* (TNF-*α*) [33]. NSAIDs and corticosteroids can reduce OA symptoms with varying improvement [31]. In this study, anti-inflammatory activity of CBE was assessed by using EPP-induced rat ear edema and carrageenan-induced rat paw edema models. EPP-induced rat ear edema model is a screening method for evaluating the anti-inflammatory activity of drugs. When EPP is applied to the rat ears, it increases vascular permeability and consequently ear edema by inducing the release of inflammatory mediators such as histamine, serotonin, kinins and PGs [34]. CBE significantly reduced rat ear edema similar to indomethacin in this screening model. The other model, carrageenan-induced rat paw edema, is considered as an acute inflammatory process and is well suited for comparative bioassay of anti-inflammatory agents [15]. The paw edema induced by carrageenan is biphasic. The initial phase, during the first hour after carrageenan injection, consists of the release of histamine and serotonin followed by bradykinin [35, 36]. In the second phase, around 3 h and lasts about 7 h after carrageenan injection, PGs play a major role [35–38]. It has been shown that the inhibition of carrageenan-induced rat hind paw edema after the 3rd h correlates well with the therapeutic effects of most effective anti-inflammatory agents [37]. CBE 250 and 500 mg/kg significantly reduced edema volume at the 1st, 3rd, and 5th h when compared with the control group but this effect was less than those of indomethacin. Thus, the inhibitory effect of CBE might be partly due to the inhibition of synthesis and/or release of those mediators, especially the COX products. Laupattarakasem et al. [7] has studied the anti-inflammatory activity of the 50% methanol extract of* C. buchanani*. They found that, in the calcium ionophore A23187-stimulated rat leukocyte model, the extract inhibits both thromboxane B_2_ and leukotriene B_4_ which are the products of COX and 5-lipoxygenase pathways, respectively. It also inhibits TNF-*α* released from lipopolysaccharide-stimulated human monocytic cell line [7]. The inhibitory effect of their extract on arachidonic metabolism supports the results of rat ear and paw edema models in our study.

Our results showed that CBE had analgesic and anti-inflammatory effects; these findings supported the traditional uses of* C. buchanani* for arthritis treatment [7–11]. The other interesting property of the agents used for treating arthritis is chondroprotective activity because the important role of the cartilage in joint function is a shock absorber during loading and joint motion [39]. In inflammatory process, the excessive production of inflammatory cytokines causes an unbalanced chondrocyte metabolism leading to the destruction of extracellular matrix and subsequent cartilage degradation [40]. We postulated that CBE had an anticytokine-induced cartilage degradation effect. One of the most important cytokines that induced cartilage degradation is IL-1*β*. Therefore, in this study, IL-1*β* was used to induce cartilage degradation. It can induce the proteolytic molecules involved in cartilage degradation such as MMP and a disintegrin-like and metalloproteinases with thrombospondin motifs (ADAMTS) [41, 42]. IL-1*β* can stimulate the production of PGE_2_ by chondrocytes. PGE_2_ could activate MMPs, aggravate joint inflammation, stimulate bone resorption, and modulate the immune response [43, 44]. IL-1*β* also decreases the anabolic activity of chondrocytes by reducing PGs and collagen synthesis [41]. In addition, the upregulation of IL-1*β* and IL-1*β*-converting enzyme have been found in human OA cartilage [45]. An intra-articular injection of recombinant porcine IL-1*β* into the pig knee joint induces the biochemical changes similar to those seen in OA [46]. After induction of cartilage degradation, CBE significantly reduced the s-GAG and HA releasing from the cartilage into the culture media while it reserved the cartilage matrix molecules such as uronic acid and collagen within the cartilage tissues. These effects were similar to those of diacerein. CBE also significantly suppressed the activity of MMP-2, a gelatinase A enzyme. MMP-2 digests gelatins and cleaves collagen type IV and V. Other substrates of gelatinase are elastin, aggrecan, and cartilage link protein [47]. These results firstly revealed the chondroprotective activity of CBE against the inflammatory cytokine-induced cartilage degradation. These may be associated with the inhibitory effects of CBE on MMPs expression via IL-1*β*-induced NF-*κ*B signaling pathway. Therefore, the further studies for evaluating the molecular mechanism of action should be performed.

The limitation of this study is that the active compound(s) of CBE has not been identified yet. However, previous studies revealed that nicotinoyl glucoside alkaloid and pyridine alkaloid (buchananine) were found in the stems of* C. buchanani* [48, 49]. The contribution of these constituents and other bioactive compounds to pharmacological activities of CBE warrants further investigation.

## 5. Conclusion

CBE shows the analgesic, anti-inflammatorily and chondroprotective activities while it has no toxicity in cartilage explants. Therefore, CBE may be useful as an alternative for the treatment of OA. Further investigations to identify the mechanisms of actions and the active compound(s) of this extract are warranted.

## Figures and Tables

**Table 1:** Effects of CBE and indomethacin on acetic acid-induced writhing response in mice (*n* = 6).

Treatment	Number of writhes	% inhibition
80% Tween	32.00 ± 2.71	—
CBE 60 mg/kg	22.00 ± 1.36^∗,††^	31.25
CBE 125 mg/kg	16.00 ± 0.96^∗,†^	50.00
CBE 250 mg/kg	15.83 ± 1.25^∗,†^	50.53
Indomethacin 5 mg/kg	9.50 ± 0.96∗	70.31

Results are mean of number of writhes ± S.E.M. ∗*P* ≤ 0.001 compared with control group. ^†^ *P *≤ 0.01 and ^††^ *P *≤ 0.001 compared with indomethacin group.

**Table 2:** Effects of CBE and indomethacin on EPP-induced ear edema in rats (*n* = 10).

Treatment	Edema thickness (mm)			
	15 min	30 min	60 min	120 min
5% DMSO in ethanol/ear	89 ± 3.79	163 ± 5.98	240 ± 8.70	169 ± 16.17
CBE 1 mg/ear	35 ± 6.20∗	64 ± 4.99∗	90 ± 9.55∗	63 ± 10.23∗
Indomethacin 1 mg/ear	20 ± 5.78∗	53 ± 11.37∗	96 ± 12.68∗	65 ± 4.28∗

Results are mean of edema thickness ± S.E.M. ∗*P *≤ 0.001 compared with control group.

**Table 3:** Effects of CBE and indomethacin on carrageenan-induced hind paw edema in rats (*n* = 6).

Treatment	Edema volume (mL)					
	1st h	% EI	3rd h	% EI	5th h	% EI
80% Tween	0.36 ± 0.05	—	0.83 ± 0.05	—	0.74 ± 0.03	—
CBE 100 mg/kg	0.18 ± 0.02	51.61	0.55 ± 0.03∗	33.78	0.57 ± 0.06	22.58
CBE 250 mg/kg	0.14 ± 0.02∗∗	60.64	0.45 ± 0.03∗	45.60	0.49 ± 0.06∗	34.28
CBE 500 mg/kg	0.12 ± 0.03∗∗	66.00	0.41 ± 0.07∗	50.37	0.40 ± 0.05∗	46.06
Indomethacin 10 mg/kg	0.06 ± 0.03∗∗	82.39	0.25 ± 0.02∗∗	70.32	0.31 ± 0.04∗∗	58.36

Results are mean of edema volume ± S.E.M. % EI is percentage of edema volume inhibition of test compound. ∗*P *< 0.05 and ∗∗*P *≤ 0.01 compared with control group.
